# Uncovering *Cyperus polystachyos* in Europe: Nomenclatural Insights and New Historical Records

**DOI:** 10.3390/plants14213270

**Published:** 2025-10-26

**Authors:** Duilio Iamonico, Filip Verloove, Sofia De Mei

**Affiliations:** 1 Department of Environmental Biology, University of Rome Sapienza, Piazzale Aldo Moro 5, 00185 Rome, Italy; demei.1928599@studenti.uniroma1.it; 2 Meise Botanic Garden, Nieuwelaan 38, B-1860 Meise, Belgium; filip.verloove@plantentuinmeise.be

**Keywords:** *Cyperus*, Italy, Pozzuoli, Tor Caldara Reserve, typification

## Abstract

*Cyperus polystachyos* Rottb. is a species primarily distributed across tropical and subtropical regions of the world, while in Europe it remains very rare, with confirmed records only from two Italian sites, i.e., Tor Caldara Natural Regional Reserve (on the southern Lazio coast, central Italy) and Ischia Island (Campania region, southern Italy), where it grows in an open habitat on sulphur-rich soils and in Hungary, along the Danube River (an historical occurrence based on a herbarium collection dated October 1891). Following a detailed examination of specimens preserved in several European herbaria, we identified a previously overlooked historical collection from Sicily, a region where the species had long been considered absent but where this specimen provides the first confirmed evidence of its historical presence. Morphologically, *C. polystachyos* is highly polymorphic, leading to the description of many taxa over the centuries. Of these, only two varieties are currently accepted, i.e., var. *polystachyos* and var. *holosericeus* (Link) C.B.Clarke. To clarify the application of these varietal names, we conducted a nomenclatural study of Rottbøll’s *C. polystachyos* and Link’s *C. holosericeus* Link (the basionym of *C. polystachyos* var. *holosericeus*). We designate a specimen housed at C (barcode C10010299), collected by König in India, as the lectotype of *C. polystachyos* and a specimen deposited at K (barcode K002543977), collected by Drummond in the United States, as the neotype of *C. holosericeus* (no original material appears to survive for Link’s name). For nomenclatural purposes, we also examined the names *C. fascicularis* Poir. and *C. scopellatus* Rich., two of the earliest names associated with *C. polystachyos*. These are lectotypified here on specimens P00644234 (Poiret’s collection) and P00254684 (Richard’s collection), respectively.

## 1. Introduction

The genus *Cyperus* L. (tribe Cypereae Dumort., subtribe Cyperinae Pax) is one of the most species-rich in the family Cyperaceae Juss., comprising over 950 species with a nearly worldwide distribution, especially in warm-temperate and subtropical regions [1,2,3,4]. The flora of Europe includes 43 taxa, 20 of which are considered alien [4].

From a taxonomic perspective, *Cyperus* is a complex genus, especially regarding the infrageneric classification at the sectional level, where several groups still require further investigation [4,5,6]. In this context, the taxonomic position of *C. polystachyos* Rottb., which is the focus of the present paper, is currently accepted in *Cyperus* sect. *Pycreus* (P.Beauv.) Griseb., following the treatment of Verloove [4] and, concerning *C. fascicularis* Poir., *C. holosericeus* Link and *C. scopellatus* Rich., POWO [3].

As part of ongoing research on the flora of the Lazio region (central Italy) [7,8,9,10,11] and on nomenclatural and taxonomic aspects of critical of Italian taxa [12,13,14,15,16], we present new data concerning *Cyperus polystachyos*, a species very rare in Europe previously known to occur only on the continental part of Italy but for which this study now confirms its historical presence also on the island of Sicily, as well as in Hungary. For nomenclatural purposes, we also investigate the names *C. fascicularis* Poir., *C. holosericeus* Link, and *C. scopellatus* Rich., three of the earliest names historically associated (and currently synonymized) with *C. polystachyos*, all published in the 18th century.

## 2. Materials and Methods

This study is based on field surveys carried out during the spring and summer of 2024 and 2025, an analysis of the pertinent literature (cited throughout the text), and the search and examination of herbarium specimens preserved at BM, DR, G, GJO, HAL, K, P, PAL, PI, PRC, RO, and TUB (acronyms following Thiers [17]).

Nomenclatural articles, as cited throughout the text, follow the International Code of Nomenclature for algae, fungi, and plants (*Madrid Code*) [18].

The names investigated are presented in chronological order based on the publication date of their protologues.

## 3. Results and Discussion

### 3.1. Typification of the Names

#### 3.1.1. *Cyperus polystachyos*

**Figure 1:**
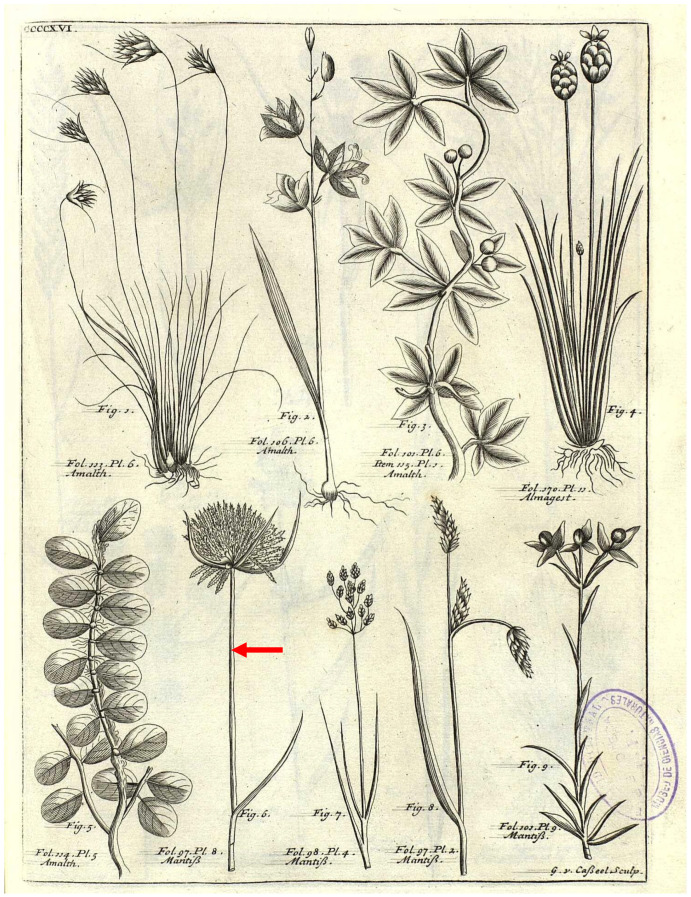
Plukenet’s illustration (Plate CDXVI, Figure 6, red arrow) of *Cyperus polystachyos*.

Rottbøll [19] (p. 21) validly described *Cyperus polystachyos*, providing a Latin diagnosis and citing an illustration by Plukenet [20] (“Table 416. Figure 6”; Figure 1), which forms part of the original material for the name in accordance with Art. 9.4 of the ICN. Additionally, the letter “K.” was indicated after the diagnosis, meaning “König” as clarified in Rottbøll’s Introduction [19] (p. 9, “Plantas, quibus in hac opella litera K. adscripta videtutur, Medicus atque Botanicus egregius Konigius indefessa industria, & lynceis oculis in itinere, quo stationem suam in regione Malabarica adiit indagavit …” = The plants to which the letter K. appears to have been assigned in this work refer to the excellent physician and botanist Konigius who, with untiring energy and eyes of lynxes, in the journey which he took to his station in the region of Malabar, traced …). This is a syntype (Art. 9.6 of ICN).

The herbarium and types of Christen Friis Rottbøll are primarily preserved at C [21], where we located a specimen (barcode C10010299) bearing a plant collected by König in India, as noted (on verso) in the handwritten annotation (“König est India …”). We consider specimen C10010299 as part of the original material for the name *Cyperus polystachyos* and designate it here as the lectotype, since its morphology closely matches both Rottbøll’s diagnosis and the current application of the name [4,22]). It is worth noting that a second specimen (barcode C10010297), available online at https://www.gbif.org/occurrence/466629932 (accessed on 15 July 2025), also belongs to König’s Herbarium, as indicated by a label on the bottom-centre of the sheet. However, because no locality is recorded and its provenance is uncertain, we exclude this specimen from consideration for lectotypification.

#### 3.1.2. *Cyperus fascicularis*

*Cyperus fascicularis* was described by Poiret [23] based on a brief diagnosis (“*Culmo triquetro*, *basi folioso*, *panicula fasciculato-capitata*”); no holotype was indicated, while a reference to an illustration by Plukenet (“pl. 416, Figure 6”) published in his *Amaltheum Botanicum* [20]. This illustration forms part of the original material for *C. fascicularis* (Figure 1). Notably, the same illustration had previously been cited by Rottbøll [19] (p. 21) in the protologue of *Cyperus polystachyos*.

Poiret’s Herbarium and types are mainly preserved at P [20], where we located a specimen (barcode P00644234) bearing an original label marked “*herb. Poiret*”. This specimen qualifies as original material for *Cyperus fascicularis* under Art. 9.4 of the ICN. An additional sheet (P00568826), available online https://plants.jstor.org/stable/viewer/10.5555/al.ap.specimen.p00568826; (accessed on 15 July 2025), bears a printed “ISOTYPE” annotation. However, its label reads “*Cyperus fascicularis. Fl. Atl. 44*|*Poiret voy.*”, referring to volume 1 of Desfontaines’ *Flora Atlantica* [24] (p. 44), published in 1798. Therefore, there is no evidence that this specimen represents original material for *C. fascicularis*, either as an isotype (Art. 9.5) or as a syntype (Art. 9.4).

All things considered, specimen P00644234 is here designated as the lectotype of *Cyperus fascicularis*, as it corresponds morphologically with Poiret’s diagnosis and aligns with the current circumscription of *C. polystachyos* [4,22]. Consequently, *C. fascicularis* and *C. polystachyos* are heterotypic synonyms, with Rottbøll’s name having nomenclatural priority (publications dates: 1772 vs. 1789).

#### 3.1.3. *Cyperus scopellatus*

*Cyperus scopellatus* was published by Richard [25] (p. 106) providing solely a Latin diagnosis (“*3-queter, angustifolius; radiis umbellae apice fasciculato-scopaeformibus; spiculis strictis, angusto-linearibus*”). Although no locality was mentioned directly in the species treatment, the provenance can be inferred as French Guiana, based on the title of the work (“CATALOGUS PLANTARUM, AD SOCIETATEM, INEUNTE ANNO 1792, E CAYENNA MISSARUM A DOMINO LE BLOND”).

According to the HUH database [21], Richard’s herbarium and types are mainly preserved at P, where we located a specimen suitable for lectotypification (barcode P00254684). This sheet contains several plants (all from the same gathering) collected by L. C. M. Richard in “*sylvurarum*”, as noted on the original handwritten label in the bottom-left corner. The specimen is part of the “Herbarium Guyanensis-Antillanum”. A more recent on the sheet reads “*Holotype de Cyperus scopellatus L. C. Rich.*”; however, since Richard [25] (p. 106) did not cite any specimen in the protologue, P00254684 cannot be considered a holotype under Art. 9.1 of the ICN. Nonetheless, it qualifies as original material and is therefore eligible for lectotypification. We designate it as the lectotype of *Cyperus scopellatus.* The specimen’s morphology is consistent with Richard’s original diagnosis and corresponds with the current circumscription of *C. polystachyos* [4,22]. As such, *C. scopellatus* and *C. polystachyos* are heterotypic synonyms, with Rottbøll’s name having nomenclatural priority (publication dates: 1772 vs. 1792).

#### 3.1.4. *Cyperus holosericeus*

*Cyperus holosericeus* was validly published by Link [26] (p. 317), who provided a diagnosis based on plants cultivated in the *Hortus Regius Botanicus Berolinensis*. Therefore, the Berlin Botanical Garden is considered the *locus classicus* for this taxon.

According to the HUH database [21], Link’s herbarium and types are primarily housed at B. However, the majority of collections at B were destroyed during World War II, as noted by many authors (e.g., [27,28,29,30]). Although additional material was searched for at BR, C, FI, H, LIV, P, PH, and W, no specimen suitable for lectotypification could be located. Consequently, in accordance with Art. 9.8 of the ICN, a neotype must be designated to fix the application of the name. We designate a specimen preserved at K (barcode K002543977), collected by T. Drummond (collection number: 454) in Texas, as the neotype of *C. holosericeus* [3]. This specimen bears a complete plant, originates from within the native distribution range of the taxon [3], and was cited by Clarke [31] (p. 55) in his taxonomic treatment of Indian species of *Cyperus*, in which he reduced *C. holosericeus* to varietal rank under *C. polystachyos*.

### 3.2. Taxonomic Notes

Over the centuries (from the 17th to the 20th century), a large number of taxa have been described for plants morphologically similar to Rottbøll’s *Cyperus polystachyos*. According to POWO [3], *C. polystachyos* s.l. currently has 39 synonyms and 22 additional nomenclatural changes, of which 32 and 14, respectively, pertain to var. *polystachyos*, and 7 and 8 to var. *holosericeus*. These figures reflect the high degree of phenotypic variability exhibited by *Cyperus polystachyos* (as highlighted by Verloove [4] who stated that this species “belongs to an unresolved assemblage of species”). While POWO [3] recognizes var. *holosericeus* as the sole accepted infraspecific taxon, some authors (e.g., [4,32]) prefer to avoid formal infraspecific classification altogether, instead treating the species broadly as polymorphic. Others (e.g., [33]) have called attention to additional forms, such as var. *brevispiculatus* F.C.How & Y.F.Deng, which may warrant separate recognition. The most comprehensive treatment of *C. polystachyos* is that of Clarke [31] who recognized nine varieties [var. *polystachyos*, var. *laxiflora* (Poir.) C.B.Clarke, var. *minor* (Cherm.) C.B.Clarke, var. *ferrugineus* Boeckeler, var. *micans* (Kunth) C.B.Clarke, var. *holosericea*, var. *paniculata* (Rottb.) C.B.Clarcke, var. *filicina* (Vahl.) C.B.Clarke, and var. *cleaverii* (Torr.) C.B.Clarke]. These were differentiated based on features such as inflorescence structure, spikelet colour and length, and the number of flowers per spikelet. In the present study, we follow POWO [3] and the Global Cyperaceae Database [34] in recognizing only var. *holosericeus* at the infraspecific level. A comprehensive global revision of this unresolved species complex remains a priority.

### 3.3. Taxonomic Treatment

***Cyperus polystachyos*** Rottb., Descr. Pl. Rar. 21. 1772 var. ***polystachyos*** ≡ *Pycreus polystachyos* (Rottb.) P.Beauv., Fl. Oware 2: 48. 1816 ≡ *Chlorocyperus polystachyus* (Rottb.) Rikli in Jahrb. Wiss. Bot. 27: 563. 1895.

**Figure 2:**
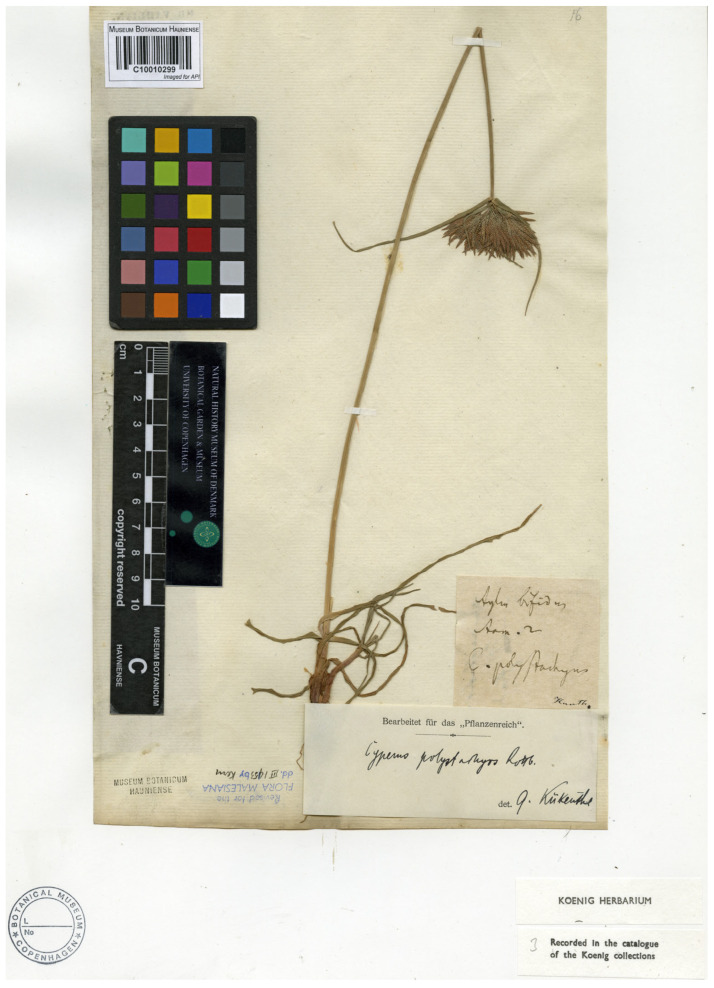
Lectotype of the name *Cyperus polystachyos* (C10010299); magnification of König’s handwritten annotation (“König est India”) on the top-right corner of the sheet. Reproduced with permission of the Natural History Museum of Denmark.

Lectotype (here designated; Figure 2):—INDIA. *s.d.*, *König s.n.* (C; C10010299!, image of the lectotype available at https://www.gbif.org/occurrence/466630012; accessed on 15 July 2025).

= *Cyperus fascicularis* Poir., Voy. Barbarie 2: 88. 1789.

Lectotype (here designated):—AFRICA. *s.d.*, *Herbarium Poiret s.n.* (P; P00644234!, image of the lectotype available at https://plants.jstor.org/stable/viewer/10.5555/al.ap.specimen.p00644234?loggedin=true; accessed on 22 October 2025).

= *Cyperus scopellatus* Rich., Actes Soc. Hist. Nat. Paris 1: 106. 1792.

Lectotype (here designated):—FRENCH GUYANA. *In sylvurarum*, *s.d.*, *Richard s.n.* (P; P00254684!, image of the lectotype available at https://plants.jstor.org/stable/viewer/10.5555/al.ap.specimen.p00254684; accessed on 24 October 2025).

− *Pycreus polystachyos* var. *typicus* Domin, Biblioth. Bot. 20(85): 416. 1915, *nom. inval.* (Art. 24.3 of ICN).

**Figure 3:**
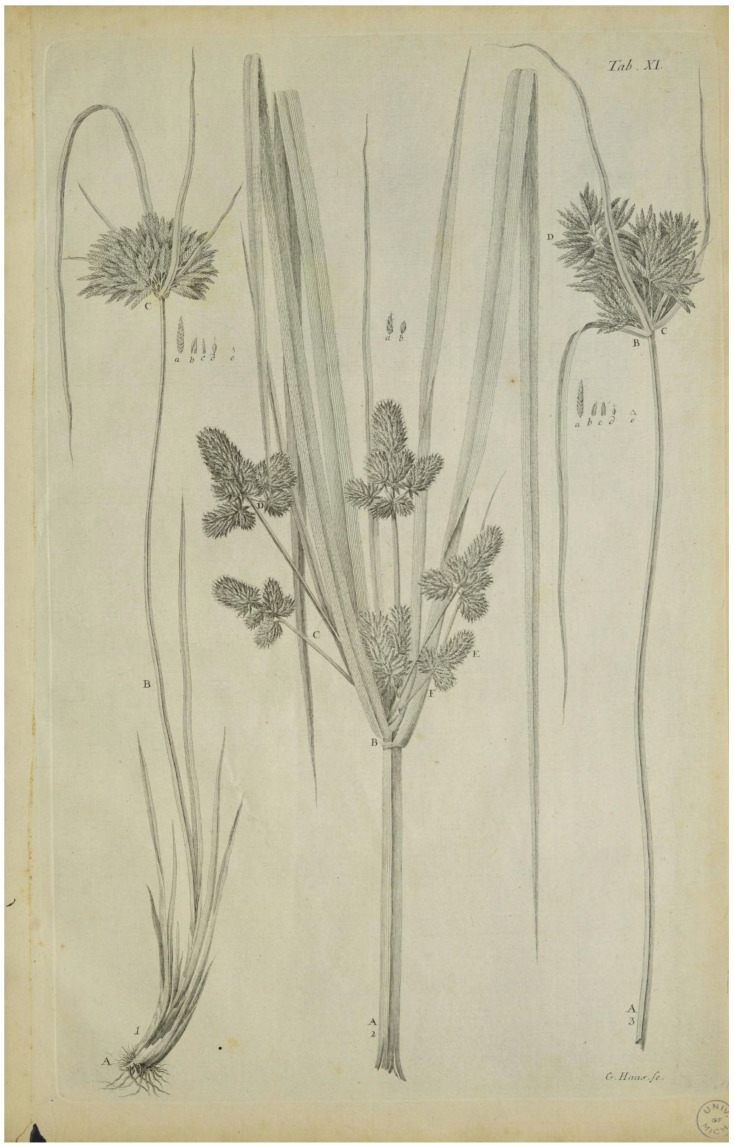
Rottbøll’s iconography (“Table XI, Figure 1”; drawing on the left) [35].

**Further iconography:** Rottbøll [35] (Figure 3).

**Figure 4:**
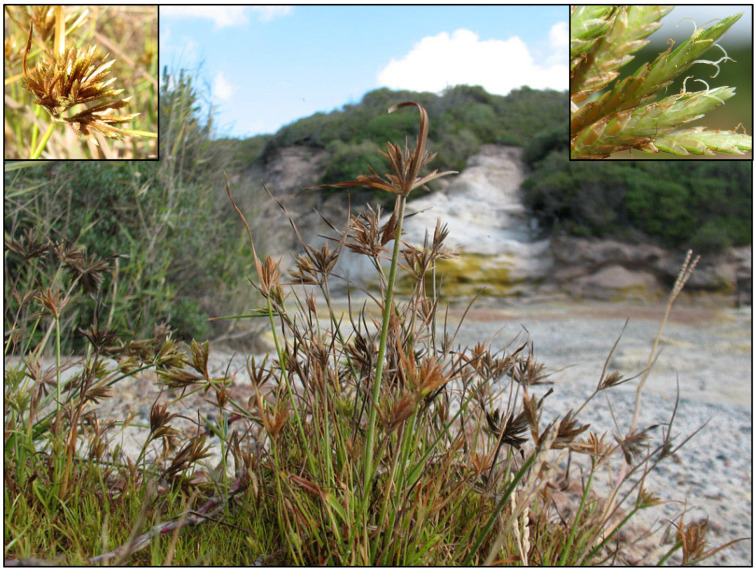
*Cyperus polystachyos* var. *polystachyos* at Tor Caldara Natural Regional Reserve (south of Lazio region, central Italy); detail of inflorescence (top-left corner of the figure) and spikelets (top-right corner of the figure). Photos by Gianluca Nicolella (licence CC BY-NC-ND 4.0).

**Distribution in Europe:** *Cyperus polystachyos* var. *polystachyos* mainly occurs in tropical and subtropical regions of the world, especially in the Southern hemisphere [3]. In Europe, however, the species is very rare and has been documented, according to literature (see, e.g., POWO [3]), from only two sites in Italy: the Tor Caldara Natural Regional Reserve (south of Lazio region, central Italy [36]; Figure 4) and Ischia Island (Campania region, southern Italy [37]), where it occurs in solfataras. Our revision of herbarium specimens led to the discovery of a previously unrecorded exsiccatum deposited at GJO (barcode GJO0052764), collected in Sicily (southern Italy) by Antonio Orsini (born on 9 February 1778, died on 18 June 1870), an Italian naturalist who contributed extensively to the floristic documentation of central and southern Italy [38]. Although the specimen lacks precise locality data, as the original label (located in the bottom-right corner of the sheet) does not specify any site, this record nonetheless confirms, for the first time, the historical presence of *Cyperus polystachyos* on the island of Sicily. It is worth noting that the species’ occurrence in Sicily has long been debated. Its presence on the island was questioned, and in 2007 [39], *Cyperus polystachyos* was officially removed from the checklist of the Italian flora for Sicily due to “erroneous records” (“segnalazioni erronee”) [40]. The discovery of this specimen thus adds a critical piece of evidence, rectifying past uncertainties.

In addition to Italian records, a specimen collected by P. Commerson along Danube River in Hungary in October 1891 has been traced and it constitutes the earliest known national record, although it should be regarded as a historical occurrence.

**Ecology and conservation status:*** Cyperus polystachyos* is a thermophilic, heliophilic, and hygrophilous species typically found in open habitats with sulphur-rich, wet soils (Figure 4). Due to its extremely limited distribution in Italy, the species has recently been assessed as Critically Endangered at the national level [41]. Major threats include groundwater extraction, increasing tourism pressure, and limited natural dispersal capacity. The old collection by A. Orsini (XIX century), which we traced at GJO, cannot be considered for the current assessment of the species in Italy. Similarly, the only record found for Hungary, represented by and old collection (year: 1891), does not take into account any IUCN assessment at European level. So, the IUCN category of Critically Endangered assessed by Fabrini and Crosti [41] for Italy can also be considered for the whole continent.

**Specimina Visa Selecta:** ALGERIA. E erbaria prope la calle, *s.d.* (XVIII century), *R.L. Desfontaines s.n.* (P; P00668506!). AUSTRALIA. Kurnell, Botany Bay, N.S.W., May 1906, *J.L. Boorman s.n.* (P; P02243363!); Saibai Island, a weed of village gardens, 21 October 1981, *Clarkson 3892* (P; P01702846!). BRAZIL. São Paulo, in einem Sumpf bei S. Paulo in Brasilien auf schwarzer, humoser Erde, 700 m u.d.M., 6 February 1908, *Rabello & Barbosa s.n.* (PI; PI026563!). CENTRAL AFRICAN REPUBLIC. Oubangui, sur gneiss humide près vill. Gboyo 100 km E. Bambari, 5 October 1927, *Tisserant 2308* (P; P00573107!). CHILE. Détroit de Magellan, *s.d.* (XVIII century), *P. Commerson s.n.* (P; P00668510!). EGYPT. In Qasr Dachel, 23 January 1874, *Ascherson 2303* (G; G00753849!). FRENCH GUYANA, In sylvurarum, s.d., Richard s.n. (P; P00254684!). HUNGARY. Danubii, October 1891, *Rochel s.n.* (K; K002453610!). INDIA. Pondichery, *s.d.*, *Couzier s.n.* (P; P00668508!). ITALY. **Campania**, Ischia, Ischia, in vaporariis, 14 January 1845, *C. Alexander s.n.* (K; K002453616!); in vaporariis, 1845, *C. Alexander s.n.* (K; K002453615!); Ischia, alla stufa d’Ischia alla temperatura di 50–70 gradi, November 1840, *s.c. s.n.* (PAL; PAL51147!); Ischia, vapours of Casamicciola Ischia, June 1855, *s.c. s.n.* (K; K002453617!); Ischia, vapours of Casamicciola Ischia, June 1855, *s.c. s.n.* (K; K002453618!); Ischia, la stufa dei Cacciuti, 1864, Bolle 559 (PAL; PAL51143!); Ischia, in terra calida (a grad 20 ad 70 C) ad vaporaria (“Fumarole” dicta) naturalia insula Inarimes, Stufa del Cacciuto prope Casamicciola, 6 September 1875, *Levier s.n.* (DR; DR061585!; Ischia, in terra calida humenti (a grad. 20 ad 70 °C) ad vaporaria naturalia (Fumarole dicta), September 1875, *Levier s.n.* (K; K002453619!); Ischia, Ins. Inarime, 1879, *Pedicino s.n.* (PAL; PAL51142!); Ischia, September 1891, *Gussone 18* (K; K002453613!); Ischia, Fumaiuole di Montecito, 3 July 1898, *Micheletti s.n.* (PI; PI026562!); Ischia, in insulae Inarimes vaporariis (60 °C) Casamicciola, 25 May 1900, *Guadagno s.n.* (GJO; GJO0052768!); Ischia, in insulae Inarimes Casamicciola, 25 May 1900, *Guadagno s.n.* (DR; DR061580!); Ischia, Fumarole dei Cacciuti, May 1900, *Guadagno s.n.* (PI; PI026561!); Ischia, Ins. neapol. Ischia locis ubi e terra erumpunt vapores calidi; temperatura terrae circiter 50°, Fumarole di Montecito, 29 September 1903, *Guadagno s.n.* (DR; DR061582!); Ischia, in herbosis humentibus prope vaporarium naturale vulgo Fumarole di Bellomo., 1 October 1907, *Guadagno 743* (K; K002453612!); Ischia, Inarime insula, in herbosis humentibus prope vaporarium naturale vulgo Fumarole di Bellomo, alt. 400 m circ., solo siliceo, 1 October 1907, *Guadagno 743* (PI; PI026559!); Ischia, Fumarole di Bellomo con le radici nel fango a 50°, 1 October 1907, *Guadagno s.n.* (PI; PI026560!); *s.d.* (XIX century), *s.c. s.n.* (K; K002453614!); Ischia, *s.d.* (XIX century), *Gussone s.n.* (GJO; GJO0052765!); Ischia, *s.d.*, *s.c. s.n.* (PAL; PAL 51140!); Ischia, si trova vicino alle fumarole di Ischia, *s.d.*, *s.c. s.n.* (PAL; PAL51141!); Ischia, ai Cacciuti sul suolo caldo alla temp. 60°, *s.d.*, *Pasquale s.n.* (PAL; PAL51144!); Ischia, alla stufa dei Cacciuti, *s.d.*, *G. Avellino s.n.* (PAL; PAL51145!); Ischia, alla stufa dei Cacciuti (unica località del Regno dove si trova), *s.d.*, *G. Avellino s.n.* (PAL; PAL51146!); **Lazio**, Metropolitan City of Rome Capital, Anzio Municipality, TorCaldaraNatural Regional Area, fumarole named “Cava Grande”, 12 June 2024, *Iamonico & De Mei s.n.* (RO); *ibidem*, 31 July 2024, *Iamonico & De Mei s.n.* (RO); *ibidem*, 18 August 2024, *Iamonico & De Mei s.n.* (RO); *ibidem*, 14 July 2025, *Iamonico & De Mei s.n.* (RO); **Sicily**, *s.d.* (XIX century), *Orsini s.n.* (GJO; GJO0052764!). LIBERIA. Grand Cape Mount, behind beach close to Robertsport. Dens low vegetation on sandy soil. 6°45.62′ N, 11°22.16′ W Alt: 1m, February 2013, *C.C.H. Jongkind 11674* (P; P00851853!). LIBYA, G. Rolph Exepdition in die lybischie Wuste, 23 January 1874, *Ascherson s.n.* (G; G00753849!); 6 April 1876, *Ascherson 534* (DR; DR061581!). MADAGASCAR. *S.d.* (XIX century), *Baron 489* (P; P00459822!); Antalavia, infrequently grazed meadow on sandy soil near to beach, 23 February 1988, *Simpson s.n.* (P; P00947600!). MAURITIUS. Ile de France, *s.d.* (XVIII century), *Commerson s.n.* (P; P00668502!); *S.d.*, *Sieber 10* (TUB; TUB007288!); *S.d.*, *s.c. s.n.* (GJO; GJO0052653!). MAYOTTE. 120 m, 12 August 1999, Pibot 506 (P; P00176566!). MARTINIQUE. Autour des ancienne plantations, 28 December 1896, Debeaux s.n. (P; P00578480!); Rivière-Salée, fossés humides, 26 April 2021, Ferlay 1195 (P; P00942131!). MEXICO. Veracruz-Llave, in palutibus prope Jalapa, September 1828, *Schiede 846* (HAL; HAL0053164!); Veracruz-Llave, in paludibus prope Jalapa, September 1828, *Schiede 846* (HAL; HAL0053163!). NEW CALEDONIA. Forête humide, 800 m, 15 April 1976, Mackee 31045 (P; P01707462!). PERU. In humidis prope Limam, *s.d.* (XVIII century), *Dombey s.n.* (P; P00668501!). PHILIPPINES. Insula Luzon, *s.d.*, *Haenke s.n.* (PCR; PRC450364!). RÉUNION. Ile de Bourbon, *s.d.* (jstoXVIII century), *Commerson s.n.* (P; P00668507!); Ins. Bourbon, *s.d.* (XIX century), *Boivin s.n.* (DR; DR061583!). SENEGAL. In paludosis prope pagum Khann ad promosta rinid, August 1827, *Leprieur s.n.* (P; P00573105!). SOUTH AFRICA. Capland, Uitenhaag, *s.d.*, *Ecklon s.n.* (DR; DR061584!). SRI LANKA. *S.d.* (XIX century), *Thwaites 800* (DR; DR061579!). THAILAND. Prov. Krabi, Ao Nang at Krabi, (98° 50′ E 08° 01′ N) Alt. 5 m, 16 July 1992, *K. Larsen*, *S.S. Larsen*, *C. Niyomdham*, *P. Sirirugsa*, *D. D. Tirvengadum*, *C. T. Norgaard 43453* (P; P00079088!). USA. North Caroline, 1823, *Schweinitz s.n.* (P; P00254554!). VIETNAM. 1500 m, 19 September 1965, *Martin 878* (P; P01786896!).

***Cyperus polystachyos*** Rottb., Descr. Pl. Rar. 21. 1772 var. ***holosericeus*** (Link) C.B.Clarke, J. Linn. Soc., Bot. 21: 55. 1884 ≡ *Cyperus holosericeus* Link, Hort. Berol. 1: 317. 1827 ≡ *Cyperus polystachyos* subsp. *holosericeus* (Link) T.Koyama, Madroño 20: 253. 1970 ≡ *Pycreus polystachyos* subsp. *holosericeus* (Link) T.Koyama in Bishop Mus. Occas. Pap. 29: 125. 1989 ≡ *Pycreus holosericeus* (Link.) Merr., Philipp. J. Sci., C 12: 231. 1917.

Neotype (here designated):—UNITED STATES. Texas, *s.d.*, *Drummond 454* (P; K002543977!, image of the neotype available at http://specimens.kew.org/herbarium/K002543977).

**Distribution area:** *Cyperus polystachyos* var. *holosericeus* has a more restricted distribution compared to var. *polystachyos*. It has been recorded in the southwestern U.S.A., eastern Mexico, Cuba, Guatemala, Costa Rica, Panama, Venezuela, Ecuador, Paraguay, eastern Brazil, western and central-southern Africa, India and southwestern Asia, and northeastern Australia [3]. To date, it has not been recorded in Europe.

**Note:*** Pycreus holosericeus* (Link.) Merr. is reported in POWO [3] and IPNI [42] as “*Pycreus holosericeus* Merr.”. However, Merril [43] explicitly proposed a new combination based on *Cyperus holosericeus*, citing it correctly as “*Pycreus holosericeus* (Link)”. Therefore, the citations provided in POWO and IPNI are incorrect.

## Data Availability

The original contributions presented in this study are included in the article. Further inquiries can be directed to the corresponding author.
